# Rheumatoid arthritis antigens homocitrulline and citrulline are generated by local myeloperoxidase and peptidyl arginine deiminases 2, 3 and 4 in rheumatoid nodule and synovial tissue

**DOI:** 10.1186/s13075-016-1140-9

**Published:** 2016-10-20

**Authors:** Sanna Turunen, Johanna Huhtakangas, Tomi Nousiainen, Maarit Valkealahti, Jukka Melkko, Juha Risteli, Petri Lehenkari

**Affiliations:** 1 Department of Anatomy and Cell Biology, Cancer and Translational Medicine Research Unit, University of Oulu, P.O. Box 5000, 90014 Oulu, Finland; 2 Division of Orthopedic Surgery, Oulu University Hospital, Oulu, Finland; 3 Division of Rheumatology, Department of Internal Medicine, Oulu University Hospital, Oulu, Finland; 4 Department of Pathology, Cancer and Translational Medicine Research Unit, University of Oulu and Oulu University Hospital, Oulu, Finland; 5 Department of Clinical Chemistry, Cancer and Translational Medicine Research Unit, University of Oulu, Oulu, Finland; 6 Northern Finland Laboratory Centre NordLab, Oulu University Hospital, Oulu, Finland

**Keywords:** Rheumatoid arthritis, Citrulline, Homocitrulline, Necrosis, Peptidyl arginine deiminase, Myeloperoxidase, Synovial tissue

## Abstract

**Background:**

Seropositive rheumatoid arthritis (RA) is characterized by autoantibodies binding to citrullinated and homocitrullinated proteins. We wanted to study the expression patterns of these disease-associated protein forms and if the rheumatoid nodule and synovial tissue itself contain biologically active levels of citrullinating peptidyl arginine deiminases 2, 3 and 4 and homocitrullination-facilitating neutrophil enzyme myeloperoxidase.

**Method:**

Total of 195 synovial samples from metatarsal joints from five ACPA/RF-positive RA patients (n = 77), synovial samples from knees of eight seropositive RA (n = 60), seven seronegative RA (n = 33) and five osteoarthritis (n = 25) patients were analyzed for citrulline and homocitrulline contents using HPLC. The location of citrulline- and homocitrulline-containing proteins, PAD 2, 3, 4 and myeloperoxidase were shown by immunostaining. Myeloperoxidase and citrulline- or homocitrulline-containing proteins were stained on Western blot.

**Results:**

Overall, necrosis was frequent in metatarsals of seropositive RA and absent in seronegative RA and osteoarthritis patients. In histological analysis, there was a significant local patterning and variation in the citrulline and homocitrulline content and it was highest in metatarsal synovial tissues of seropositive RA patients. We found peptidyl arginine deiminase 2, 3 and 4 in the lining and sublining layers of intact synovial tissue. Myeloperoxidase was found locally around necrotic areas. The tissues with necrosis contained the highest levels of citrulline and homocitrulline.

**Conclusions:**

Rheumatoid nodules and synovia contain significant amount of PAD2, 3 and 4 and myeloperoxidase enzymes. These enzymes could explain the levels of citrulline and homocitrulline in seropositive RA synovial and rheumatoid nodule tissues especially around necrotic tissue.

## Background

Rheumatoid arthritis (RA) is a chronic autoimmune disease that is characterized by painful inflammation of synovial joints. RA joint symptoms are characterized by symmetric inflammation of joints and change in synovial extracellular matrix, usually beginning in the metacarpal or metatarsal joints. The local findings include a pannus formation, i.e., invasive synovitis, formation of rheumatoid nodules and even pseudotumors of the synovial membrane and influx of inflammatory cells. The pathological process leading to RA is not fully understood. Antibodies binding to citrullinated proteins (ACPA) are a rheumatoid arthritis-specific finding that can precede clinical disease onset by several years [1], which suggests that these antibodies and their antigens could play a role in the disease pathogenesis. In addition to the joint tissues, citrullination in the lungs in response to smoking has been suggested as an inducing event (reviewed in [2]). Five different isoenzymes of citrullinating peptidylarginine deiminases (PADs) are found in humans. Of these, PAD2 and PAD4 have been shown to be present in RA synovial tissue [3]. PAD3 expression has been connected to tissue damage in chick embryonic neural stem cells [4]. However, so far the exact molecular mechanism in pathogenesis and the anatomical origin of these citrullinated and homocitrullinated proteins is not known. To elucidate these processes we selected PADs 2, 3 and 4 and myeloperoxidase as target antigens for the immunological stainings in this study.

The ACPA antigens identified so far are proteins occurring naturally in the human body. In RA, citrullinated fibrin, vimentin and histones are the focus of interest and are considered possible autoantigens for citrulline-binding antibodies [5]. Fibrin deposits are common in inflamed synovium, histone citrullination is present in extracellular trap structures created by neutrophils [6, 7] and vimentin is citrullinated during apoptosis. It has also been shown that in cell culture conditions citrullination of fibronectin alters synovial fibroblast behavior and may affect how these cells adhere to and invade the joint [8]. Since ACPA may develop years before disease onset, the processes leading to antibody development are an obvious subject of investigation. Recently, also antibodies binding to carbamylated [9] and specifically to homocitrullinated sequences have been found in RA sera [10], but also processes leading to formation of these antibodies are unknown. It has been suggested that ACPA and carbamylated protein-binding antibodies are distinct systems [11] although overlapping of the presence of both of these antibodies is frequent in RA [9, 10].

Citrulline and homocitrulline in proteins are products of different posttranslational modifications (arginine citrullination and lysine carbamylation respectively) but have similar effects on protein structure and may create epitopes that induce autoantibody responses. The formation of citrulline and homocitrulline contributes to protein immunogenity [12, 13]. Antibodies binding to citrulline- and homocitrulline-containing antigens contain partly overlapping binding sites in RA sera [9, 10, 14] and in animal models [13] suggesting some relationship between these two modifications in RA.

Lately, the role of neutrophils in RA has raised interest as neutrophil extracellular trap (NET) formation has been suggested as a source of citrullinated antigens in synovial fluid and pannus [7]. Myeloperoxidase, an enzyme that is present in the granules of neutrophils, has a function in host defense. Myeloperoxidase uses negatively charged ions (for example iodine, chloride and cyanate) [15, 16] to distort, for example, bacterial proteins to inhibit their function. The addition of cyanate to lysine side chain creates homocitrulline, which makes NETs interesting also in respect of origin of homocitrulline-binding antibodies.

We have already shown that citrulline and homocitrulline could be found simultaneously in the synovial tissues of a single ACPA-positive RA patient [17]. In this sequential study we show that the synovial tissues of seropositive RA patients are characterized by necrotic areas that contain high levels of citrulline and homocitrulline and PAD and myeloperoxidase enzymes. We also show the presence of myeloperoxidase in the necrotic areas. Necrotic areas are more frequent in the metatarsal than in the knee synovial tissues in seropositive RA patient tissues and absent in seronegative RA and in osteoarthritis (OA) patient synovial tissues.

## Methods

### Subjects

Synovial tissues of feet were obtained from five RA patients [RA+ metatarsophalangeal (MTP) group, patients 20–25] with a severe destructive arthritis and deformity of foot that were subjected to corrective orthopedic surgery (four females and one male, age 64.8 ± 11.7 years). All of these subjects fulfilled the diagnostic criteria of the American College of Rheumatology for RA [18]. All had a seropositive disease meaning that they had an elevated level of rheumatoid factor (RF) and/or citrulline peptide antibodies (ACPA). One patient had a biological therapy with a tumor necrosis factor alpha (TNF-α)-blocker, the others used conventional medication [disease-modifying antirheumatic drugs (DMARDs)].

Synovial samples of knees were obtained from patients that underwent a total knee replacement. We compared the findings of four osteoarthritis (OA, patients 1–4) with seven seronegative RA (RA-, patients 5–11) and eight seropositive RA (RA+, patients 12–19) patients. All RA patients fulfilled the diagnostic criteria of the American College of Rheumatology for RA [18]. Duration of the disease with patients with RA was 21 years as mean. RA patients with a seropositive disease were all females, age 66.7 ± 7.15 years and the others with a seronegative disease were six females and one male, age 70.0 ± 5.7 years. Patient number 6 had borderline RF level and patient number 10 had juvenile RA. Eleven of all the RA patients were using DMARDs and two patients DMARDs + a TNF-α blocker and two patients (with a seronegative disease) were not using any DMARDs because of an improved disease course. Five patients with osteoarthritis included three females and two males, age 72.6 ± 1.9 years. C-reactive protein (CRP) levels were similar in all patient groups, mean < 7 in OA knee, RA+ knee and RA+ MTP, and 8.6 in RA- knee. Seropositive RA patient number 17 had a CRP level of 17 at the time of the operation and seronegative RA patients 7, 9 and 10 had CRP levels at 13, 14 and 16 respectively.

Rheumatoid nodule tissues were collected beneath MTP4 and MTP5 and from an olecranon bursa.

### Anti-CCP

Antibodies binding to citrullinated proteins were measured by automated EliA-CCP (Phadia, Thermo Fisher Scientific, Uppsala, Sweden).

### Rheumatoid factor

Immunoglobulin (Ig)M-class rheumatoid factor levels were measured from patient sera using an automated analyzer Advia 1800 (Siemens Healthcare, Erlangen, Germany).

### Antibodies binding to citrulline- and homocitrulline-containing type I and II collagen telopeptides

All available sera were tested by enzyme-linked immunosorbent assay (ELISA) as described before [10] for binding of IgG to citrullinated and homocitrullinated type I and II collagen carboxyterminal telopeptides under standard conditions, and the binding was inhibited by addition of soluble antigens to a final concentration of 100 μg/ml. The two biotinylated peptides corresponding to the carboxyterminal telopeptide of the α1 chain of human type I collagen (NeoMPS, Strasbourg, France) represent amino acids 1193–1218, and the two biotinylated peptides corresponding to the α1 chain of human type II collagen represent amino acids 1217–1241, counted from the amino terminus in the pro α1 chains of type I or type II procollagen, respectively. The synthetic peptides that were used as inhibitors in the ELISA represent carboxyteminal ends of these parent peptides. The peptide sequences have been described before [10]. In each serum, the difference between binding with and binding without inhibition was calculated for all peptide pairs.

### Detection of protein-bound citrulline and homocitrulline on high-performance liquid chromatography (HPLC)

Five approximately 10-mg (wet weight) samples were cut from each tissue. Seventy-seven tissue specimens were analyzed from seropositive RA metatarsal tissues, 60 from seropositive RA knees, 33 from seronegative RA knees and 20 from OA knees. The samples were subjected to extensive dialysis at 4 °C, 16 h against 0.2 M NH_4_HCO_3_, pH 7.4 to separate free amino acids from protein-bound ones and freeze-dried, then rehydrated in dH_2_O, freeze-dried and hydrolyzed in 6 M HCl at 110 °C for 16 h and freeze-dried. The samples were chemically modified and analyzed on HPLC as reported previously [13]. The levels of citrulline and homocitrulline in the samples were calculated from manually integrated peak area using a calibration series of citrulline and homocitrulline.

### Statistical analysis

The peak areas of citrulline and homocitrulline content between tissues (n = 195 divided in four groups) were assessed by Kruskal-Wallis test using Analyse-It version 2.26 for Excel 12+. The differences were considered significant at *p* < 0.05.

### Western blot

Treatment of samples: tissue samples from the rheumatoid nodules were divided into capsule and necrotic inner mass, homogenized in phosphate-buffered saline (PBS) containing Pierce protease inhibitor cocktail (88666, Thermo Fisher Scientific). The samples were fractionated by centrifugation to soluble and precipitate fractions. Protein concentrations were determined by the Bradford method. Both fractions were subjected to collagenase I digestion (5273 CLSPA, Worthington Biochemical, Lakewood, NJ, USA, 1:50 to protein concentration of digested sample in 0.2 M NH4HCO3 pH 7.4 overnight at +37 °C with agitation) and parallel samples to DNase I digestion (DNaseI, DN25, Sigma-Aldrich, St. Louis, MO, USA, in 50 mM Tris-HCl, 10 mM MgCl_2_ pH 7.5 for 75 minutes at +37 °C with agitation). For demonstration of citrulline- and homocitrulline-containing proteins, 50 μg of each sample and 5 μg of carbamylated albumin were run on 10 % Bis-Tris SDS-PAGE and blotted on nitrocellulose. The samples were chemically modified with 2,3-butanedione and antipyrine in acidic conditions [13] and modified citrulline and homocitrulline were detected with chemical adduct recognizing rabbit polyclonal antibody KS350 and horseradish peroxidase (HRP)-labelled anti-rabbit IgG secondary antibody. The staining was visualized on ECL film by chemiluminesence. For myeloperoxidase staining, lysate from peripheral blood neutrophils was used as a positive control and lysate from peripheral blood mononuclear fraction was used as a negative control. Blot was stained with myeloperoxidase antibody (A0398, Dako, Glostrup, Denmark) and detected with Alexa-Fluor 680 anti-rabbit IgG (A21109, Molecular Probes, Eugene, OR, USA) and visualized with an Odyssey IR imager and Image Studio™ Software (LI-COR Biosciences, Bad Homburg, Germany).

### Immunohistochemistry

After collection the tissue pieces were kept -70 °C, fixed in formalin and embedded in paraffin, 3.5-μm sections were mounted on glass slides and stained with hematoxylin and eosin (H&E). For immunostaining endogenous peroxidases were blocked with Dako REAL Peroxidase-Blocking Solution (S2023, Dako). Citrulline and homocitrulline were stained using anti-peptidyl-citrulline, clone F95 mouse monoclonal antibody 1:250 (MABN328, EMD Millipore Corp, Billerica, MA, USA) and PAD4 with anti-human PAD4 antibody 1:300 (ab59965, Abcam, Cambridge, MA, USA), after heat-induced antigen retrieval in citrate buffer pH 6. PAD2 enzyme was stained with anti-human PAD2 antibody 1:250 (ab16478, Abcam) and PAD3 with 1:50 (ab183209, Abcam) after heat-mediated antigen retrieval in Tris-EDTA, pH 9 and myeloperoxidase without antigen retrieval with anti-human myeloperoxidase antibody 1:5000 (A0398, Dako). The immunostainings were detected using Dako REAL EnVision Detection system, Peroxidase/DAB+, Rabbit/Mouse (K5007, Dako) and counterstained with Mayer hematoxylin.

Double stainings of PAD3/KP1 and PAD3/F95 were conducted after HIER in pH 9 Tris-EDTA, PAD3 1:25, KP1 1:1000 and F95 1:25 overnight at +4 °C. PAD4/KP1 and PAD4/F95 were conducted after HIER in pH 6 citrate PAD4 1:50, KP1 1:1000 and F95 1:50 overnight at +4 °C. KP1 was detected with Alexa Fluor 488 anti-mouse IgG (A11029, Molecular Probes), F95 with Alexa Fluor 488 anti-mouse IgM μ chain (A21042, Molecular Probes) and PAD3 and PAD4 with Alexa Fluor 680 anti-rabbit IgG (A21109, Molecular Probes). Nuclei were stained with 1:500 Hoechst in PBS for 5 minutes.

## Results

### Citrulline and homocitrulline analyses

**Fig. 1:**
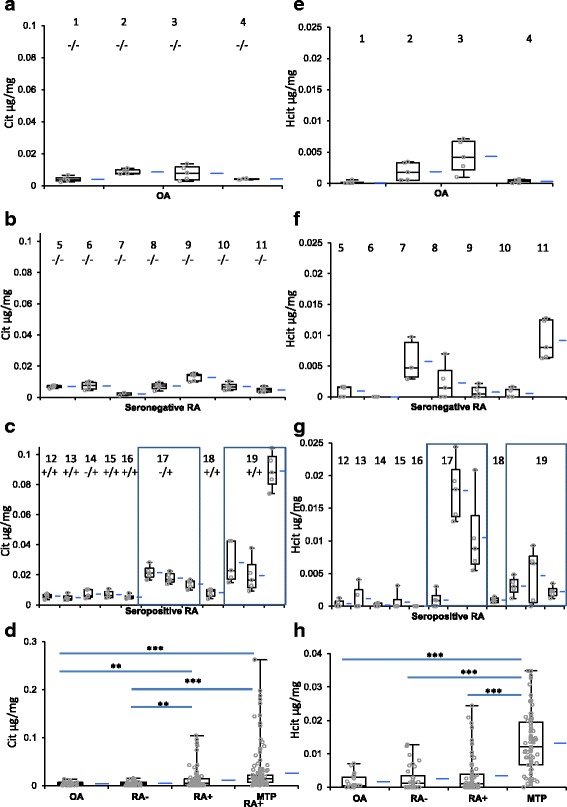
Citrulline and homocitrulline contents of synovial tissues. We compared the findings of four osteoarthritis (OA, patients 1–4) eight seropositive RA (RA+) with seven seronegative RA (RA-, patients 5–11) and eight seropositive RA (RA+, patients 12–19) patients. Statistically significant differences are indicated (^**^ *p* < 0.01, ^***^ *p* < 0.001). Patient RA serology is indicated as CCP/RF under the patient indicator. Panels A and E represent HPLC analysis results for OA, B and F seronegative RA, C and G seropositive RA knee synovial tissues. Panels D and H summarize the HPLC analysis data, including analyses of metatarsal tissue shown in detail in Fig. 2. Panels A to D represent citrulline and panels E to H homocitrulline contents shown as μg/mg of wet weight tissue. The boxplot shows the median and interquartile range (IQR) and the whiskers 95 % confidence interval (95 % CI), mean is shown as the *blue line* beside the boxplot. Individual measurements are shown as *circles. MTP* metatarsophalangeal, *OA* osteoarthritis, *RA* rheumatoid arthritis

**Fig. 2:**
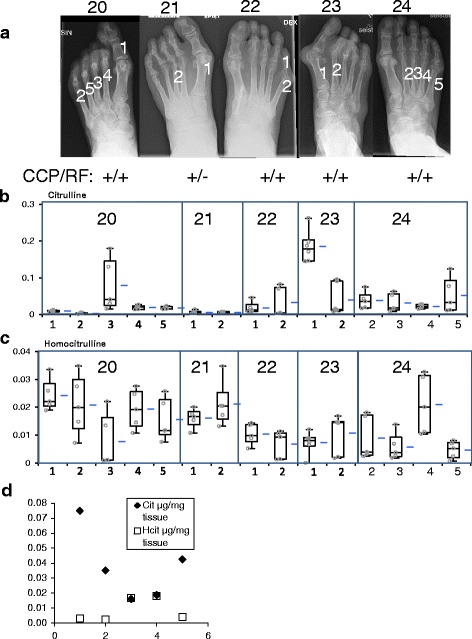
Citrulline and homocitrulline content of seropositive RA metatarsal synovial tissues. Seropositive RA metatarsal synovial tissues contained the highest levels of citrulline and homocitrulline. **a** The samples were collected from locations shown in AP foot X-rays and each tissue specimen was analyzed in five parallel analyses for (**b**) citrulline and (**c**) homocitrulline contents shown as μg/mg of wet weight tissue. The boxplot shows the median and interquartile range (IQR) and the whiskers 95 % confidence interval (95 % CI), mean is shown as the *blue line* beside the boxplot. Individual measurements are shown as *circles*. **d** Patient 24 sample 2, citrulline and homocitrulline contents represented for each individual analysis. *CCP* cyclic citrullinated peptide, *RF* rheumatoid factor

In the HPLC analyses, citrulline and homocitrulline could be found in all studied tissues (Figs. 1 and 2). The highest citrulline and homocitrulline levels were found in the seropositive RA patients’ metatarsal synovial tissues/rheumatoid nodules from this area (mean Cit 0.039 μg/mg wet weight tissue, mean Hcit 0.013 μg/mg wet weight tissue), followed by seropositive RA patients’ knee synovial tissues (mean Cit 0.019 μg/mg, mean Hcit 0.004 μg/mg). The levels of citrulline and homocitrulline in seronegative RA (mean Cit 0.007 μg/mg, mean Hcit 0.003 μg/mg) and in OA (mean Cit 0.007 μg/mg, mean Hcit 0.002 μg/mg) patients’ synovial tissues were comparable with one another and significantly lower than in seropositive RA tissues. There is about a tenfold difference in the mass amount of citrulline compared to homocitrulline, citrulline being more abundant. Overall level of carbamylation is higher than the level of homocitrulline, since homocitrulline is just one of the products of carbamylation. Homocitrulline is also only one of the products modifying protein structure as a result of myeloperoxidase activity. When several pieces from the same synovial tissue were analyzed, levels of citrulline and homocitrulline varied significantly in different areas of the same tissue. Interesting patterns between citrulline and homocitrulline were found in some of the RA patient samples. When a sample contained a high level of citrulline the level of homocitrulline was low and vice versa (Fig. 2d). This phenomenon could not be detected in other than seropositive RA tissue samples.

**Fig. 3:**
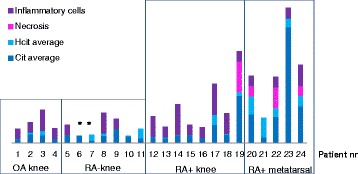
Citrulline and homocitrulline content averages combined with necrosis and inflammatory score. Inflammatory cell infiltration and necrosis were scored from 0 to 3 by tissue area. *Asterisk* indicates that no histology data is available for patients 6 and 7. Citrulline and homocitrulline contents were measured from parallel samples to histology analyses. *OA* osteoarthritis, *RA* rheumatoid arthritis

In the Kruskall-Wallis test the differences in citrulline content between groups were significant between seropositive RA knee and seronegative RA knee tissues (*p* = 0.005), seropositive RA knee and OA knee tissues (*p* = 0.002), seronegative RA knee and seropositive RA metatarsal tissues (*p* < 0.001) and OA knee and seropositive RA metatarsal tissues (*p* < 0.001). The difference between seropositive RA knee and seropositive RA metatarsal tissues (*p* = 0.323) was not significant and there was no difference between the citrulline content of seronegative RA knee and OA knee tissues (*p* = 1.000). When homocitrulline content were compared we found significant differences between seropositive RA metatarsal tissues compared to all knee tissue groups (*p* < 0.001 in all comparisons), but the knee tissues did not differ from each other in homocitrulline content (*p* = 1.000 in all comparisons). The difference in homocitrulline content was in line with the difference in the presence of necrotic areas in the tissue sections. Citrulline and homocitrulline content of the tissue combined with inflammatory infiltrate and presence of necrosis in the tissue showed cumulative effect (Fig. 3)

.

**Fig. 4:**
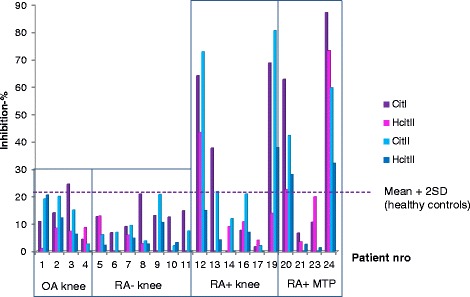
Inhibition-ELISA analysis of serum antibodies binding to citrulline- and homocitrulline-containing type I and type II collagen telopeptides. Results are shown as percentage of inhibition in standard conditions. Mean +2 SD for 72 healthy control sera is indicated by the *dotted line. MTP* metatarsophalangeal, *OA* osteoarthritis, *RA* rheumatoid arthritis

Significant levels of specific antibodies binding to citrulline- and homocitrulline-containing type I and II collagen telopeptides were found only in ACPA-positive RA patients (Fig. 4). Both citrulline- and homocitrulline-binding antibodies were elevated in the same patients.

### Western blot

**Fig. 5:**
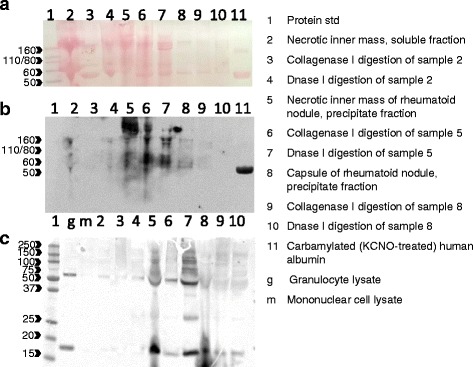
Western blot analysis of citrulline- and homocitrulline-containing proteins and myeloperoxidase in rheumatoid nodule protein samples (**a**) Ponceau S protein staining of rheumatoid nodule protein samples, (**b**) modified citrulline and homocitrulline staining of same samples, (**c**) myeloperoxidase staining. Tissue samples from rheumatoid nodule were divided into capsule and necrotic inner mass. The samples were fractionated by centrifugation to soluble and precipitate fractions. Both fractions were subjected to collagenase I and DNase I digestions, for detailed description, see Methods

In the western blot analyses (Fig. 5) citrulline- or homocitrulline-containing proteins were found mostly in the precipitate fraction of the necrotic inner mass of the rheumatoid nodule. When the sample was subjected to collagenase I digestion, some of the citrulline- or homocitrulline-containing large protein aggregates were dissolved and the citrulline and homocitrulline (KS350) staining moved more to the lower molecular weight proteins. DNase I digestion released most of the high molecular weight aggregates suggesting the presence of DNA-bound citrullinated or homocitrulline-containing proteins in the necrotic inner mass of the rheumatoid nodule. The strongest myeloperoxidase staining was also detected in the precipitate fraction of the necrotic inner mass of the rheumatoid nodule.

### Histology

In the basic H&E staining, both RA and OA synovial tissues consisted of dense, mostly mature connective tissue and infiltrated inflammatory cells. More adipose tissue was found in the OA synovial samples, and the synovial lining layer was thicker in the RA samples. One of the seropositive RA knee synovial tissues was mostly necrosis (patient 19), and in the metatarsal joint synovial tissues of seropositive RA patient’s necrosis could be found in three out of five samples (Fig. 3).

**Fig. 6:**
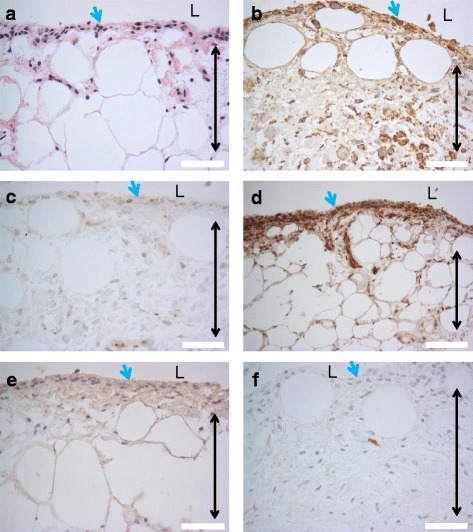
OA synovial tissue histology. **a** H&E, (**b**) citrulline and homocitrulline (F95), (**c**) PAD2, (**d**) PAD3, (**e**) PAD4 and (**f**) MPO staining. The scale bar indicates 50 μm. *L* lumen of synovial cavity, *blue arrow* synovial lining layer, *double-headed arrow* synovial stroma

**Fig. 7:**
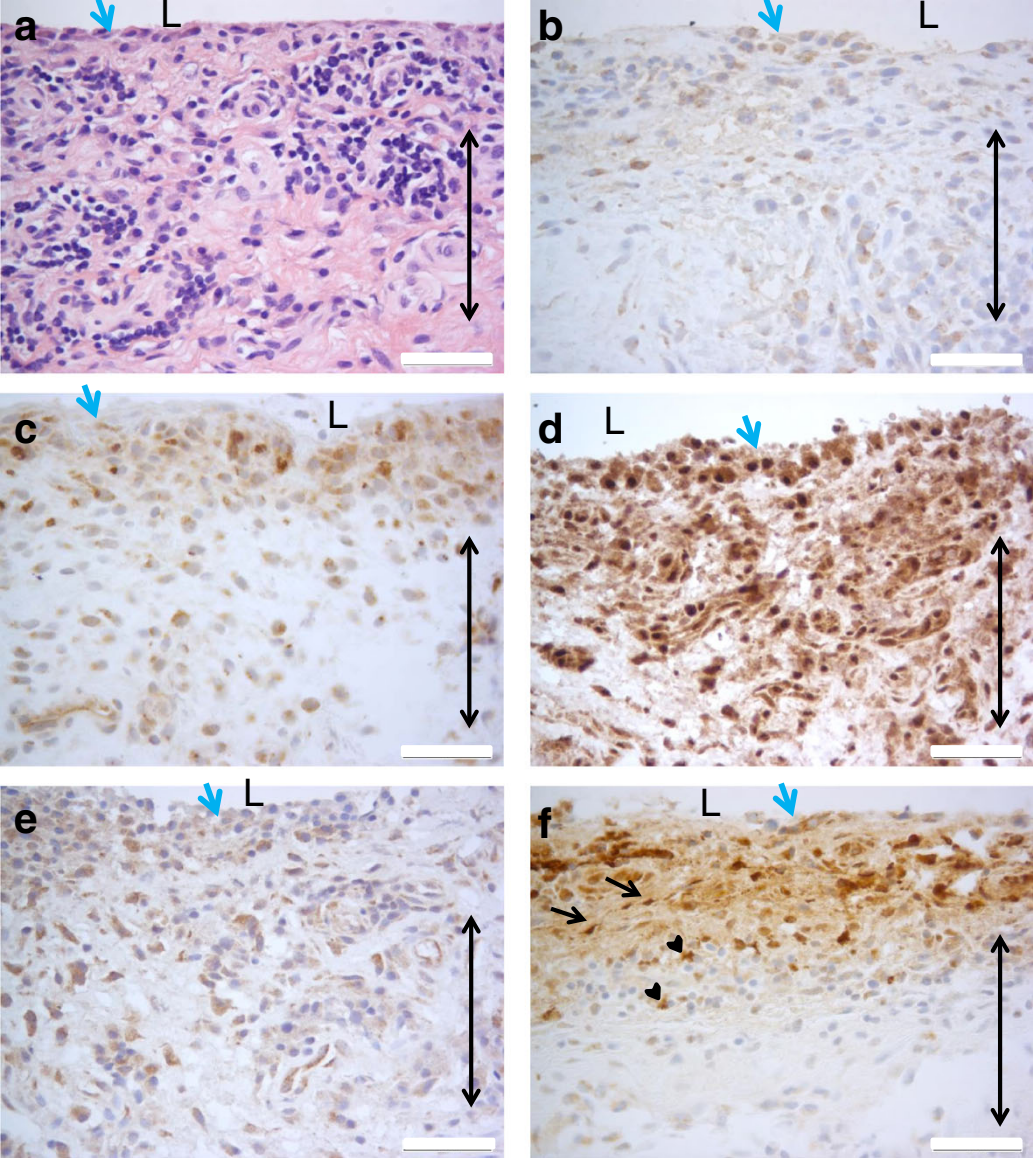
RA synovial tissue histology. **a** H&E, (**b**) citrulline and homocitrulline (F95), (**c**) PAD2, (**d**) PAD3, (**e**) PAD4 and (**f**) MPO staining. The scale bar indicates 50 μm. *L* lumen of synovial cavity, *blue arrow* synovial lining layer, *double-headed arrow* synovial stroma, *black arrow* intact neutrophil, *arrowhead* disrupted neutrophil

**Fig. 8:**
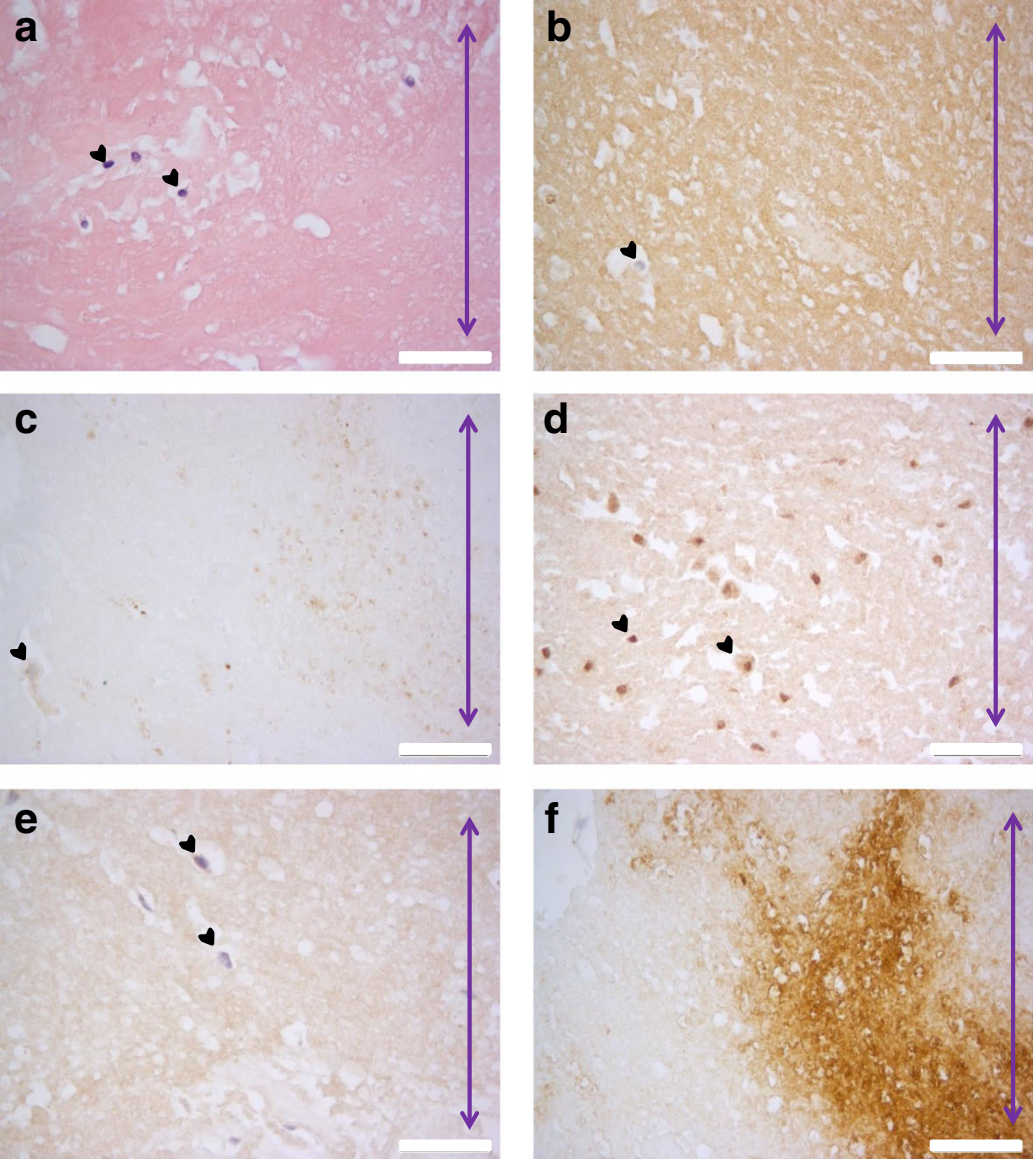
Histology of necrotic RA synovial tissue consisting of fibrinoid necrosis and sporadic intact cells. **a** H&E, (**b**) citrulline and homocitrulline (F95), (**c**) PAD2, (**d**) and PAD3, (**e**) PAD4 and (**f**) MPO staining. The scale bar indicates 50 μm. *Arrowhead* intact cells, *purple double-headed arrow* fibrinoid necrosis

**Fig. 9:**
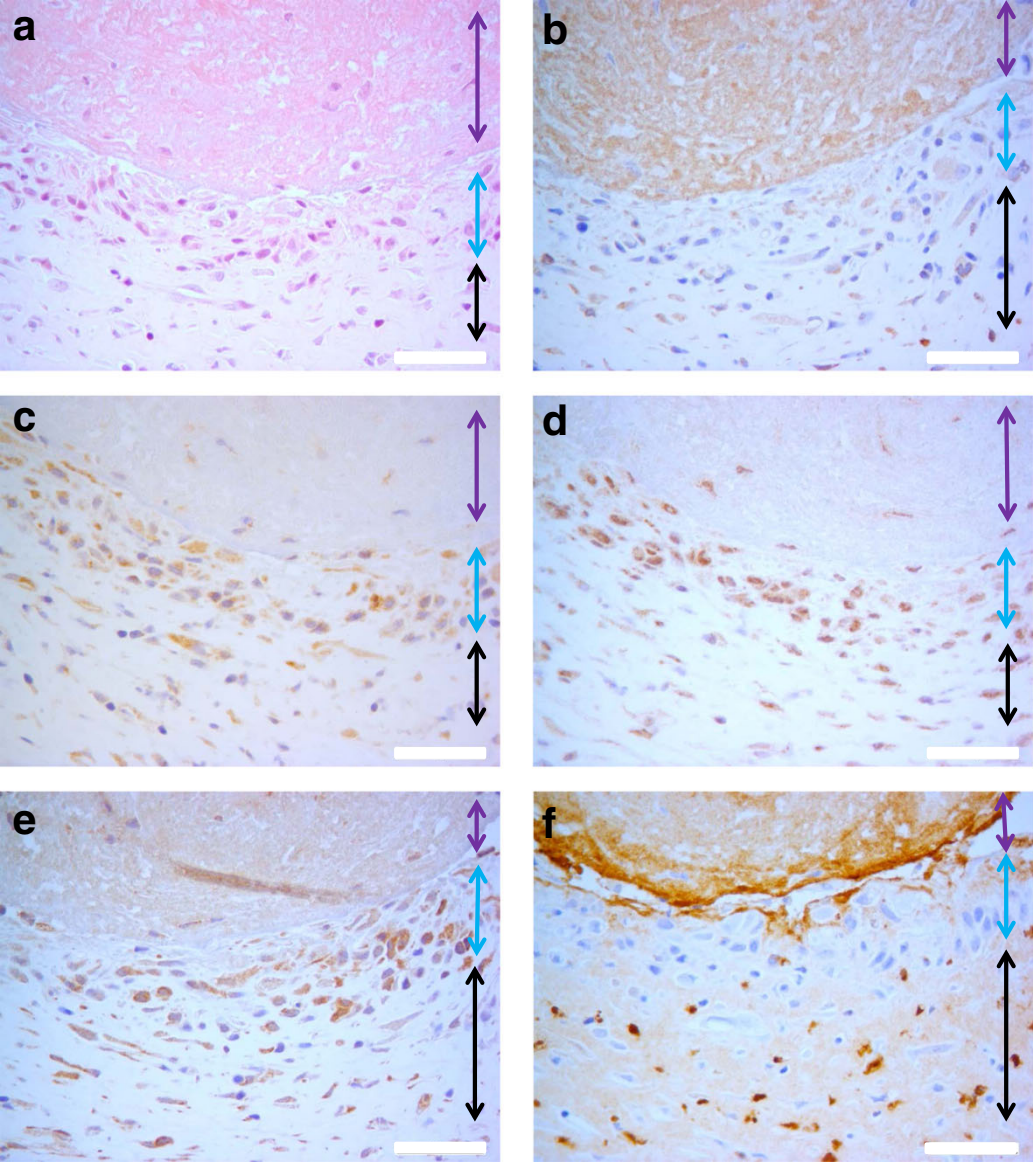
Histology of rheumatoid nodule. **a** H&E, (**b**) citrulline and homocitrulline (F95), (**c**) PAD2, (**d**) PAD3, (**e**) PAD4 and (**f**) MPO staining. The scale bar indicates 50 μm. *Purple double-headed arrow* fibrinoid necrosis, *blue double-headed arrow* palisading histiocytes, *black double-headed arrow* stromal cells

**Fig. 10:**
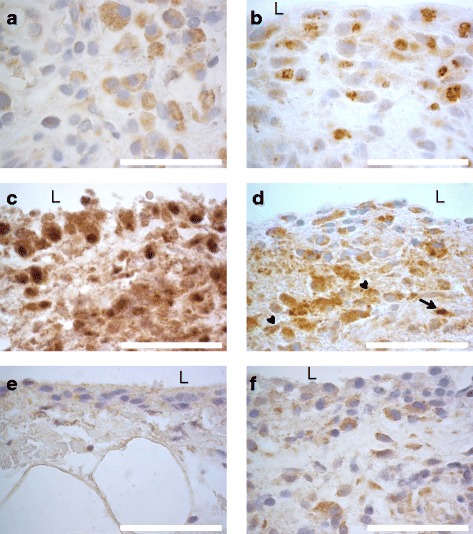
Cellular localization of (**a**) F95 in RA, (**b**) PAD2 in RA, (**c**) PAD3 in RA, (**d**) MPO in RA, (**e**) PAD4 in RA and (**f**) PAD4 staining in OA. The scale bar indicates 50 μm. *L* lumen of synovial cavity, *black arrow* intact neutrophil, *arrowhead* disrupted neutrophil

**Fig. 11:**
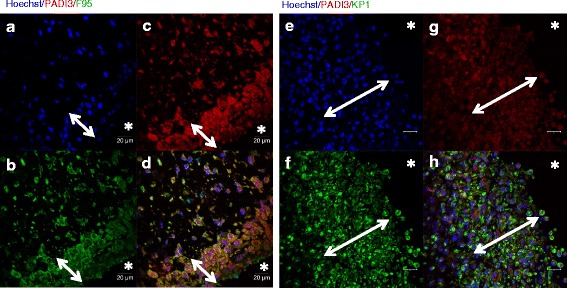
PAD3 (**c**, **g**) co-localization with citrulline- and homocitrulline-binding F95 antibody (**b**) and macrophage marker KP1 (CD68) (**g**) in the intact tissue adjacent to necrosis in rheumatoid nodule. PAD3 HIER was performed in pH 9 Tris-EDTA. PAD3 and F95 bound abundantly to the cytoplasm of cells adjacent to necrosis (**d**). PAD3 co-localized with KP1 in the cells adjacent to necrosis (**h**). *Asterisk* necrosis, *double-headed arrow* palisading histiocytes

**Fig. 12:**
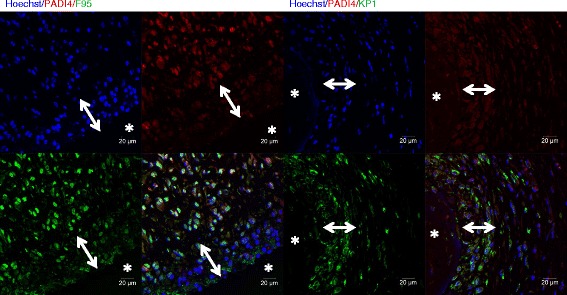
PAD4 co-localization with citrulline- and homocitrulline-binding F95 antibody and macrophage marker KP1 (CD68) in the intact tissue adjacent necrosis in rheumatoid nodule. PAD4 HIER was performed in pH 6 citrate buffer. PAD4 and F95 bound abundantly to the nucleuses and somewhat to the cytoplasm of cells surrounding the necrotic area. No nuclear staining was detected in the cells adjacent to necrosis. PAD4 co-localized with KP1 in the cells surrounding necrosis. *Asterisk* necrosis, *double-headed arrow* palisading histiocytes

The localization of citrulline- and homocitrulline-containing proteins, PAD2, PAD3, PAD4 and myeloperoxidase enzymes was defined by immunostaining (Figs. 6, 7, 8, 9 and 10). F95 antibody recognizing both citrulline and homocitrulline [19] stained strongly the fibrinoid extracellular matrix of necrotic RA synovial tissue (Fig. 8b) and the synovial lining layer and the endothelium of the small blood vessels in intact RA synovial tissue. The cell-free necrotic tissue areas were completely stained with the F95 antibody (Figs. 8b and 9b) showing citrulline- or homocitrulline-containing proteins in these areas. In intact tissue, F95 staining localized to cells with macrophage-like appearance (large, rounded nucleus and abundant cytoplasm), some fibroblast-like stromal cells, extracellular matrix and endothelium of blood vessels (Figs. 6b, 7b and 10a). PAD2 staining in the synovial lining cells was localized in the vicinity of the nuclear membrane, suggesting localization to the Golgi area (Figs. 5c, 7c, 9c and 10b). In the necrotic tissue PAD2 was sporadic, possibly localizing in areas with cell remnants co-localizing with disperse myeloperoxidase staining (Fig. 8c). PAD3 staining in both RA and OA tissue was both nuclear and cytoplasmic in the cells of the synovial lining layer and endothelium of blood vessels (Figs. 6d, 7d, 10c). In necrotic areas of RA tissue, PAD3 was staining diffusely to the extracellular matrix and in the nucleus and cytoplasm of the few intact cells (Figs. 8d and 9d). In RA tissue (Fig. 10f), but not in OA (Fig. 10e), cytoplasmic PAD4 expression was detected in macrophage-like synoviocytes both in the synovial lining and sublining layers and endothelium of blood vessels, also few positive cells were seen in deeper layers (Figs. 6e, 7e and 9e). In necrotic areas, PAD4 stained diffusely to the matrix (Figs. 8e and 9e). In the intact tissue adjacent to necrosis, PAD3 and PAD4 staining co-localized to the cells positive for macrophage marker KP1 (Figs. 11 and 12). In seropositive RA, tissue myeloperoxidase staining was localized in the extracellular matrix of the synovial lining layer and some intact small cells (neutrophils) and in local dispersed areas (disrupted neutrophils) (Fig. 7f). In necrotic tissue, diffuse myeloperoxidase was found in the matrix, but not in intact cells, suggesting release of myeloperoxidase from neutrophils (Fig. 8f). Also intact neutrophils were found in the intact tissue adjacent to necrosis and in the synovial lining layer (Figs. 9f and 10d). No myeloperoxidase staining was found in OA tissue (Fig. 6f).

## Discussion

Since ACPA and carbamylated protein antibodies are a sign of disease severity and a predictor of joint damage in RA, we wanted to study the expression patterns citrulline- and homocitrulline-containing proteins in tissue from RA and control patients. We had the opportunity of collecting and comparing RA synovial tissues from metatarsal and knee joints and found interesting differences between these two anatomical locations both in histology and citrulline and homocitrulline formation. Expression of citrullinating peptidyl arginine deiminases 2, 3 and 4 and homocitrullination-facilitating neutrophil enzyme myeloperoxidase were studied, because these enzymes contribute to formation of these altered proteins.

The staining of PAD2, PAD3 and PAD4 enzymes localized to the lining in OA and both the lining and sublining layers of synovial tissue in RA. PAD2 was found in the synovial lining and sublining layers and endothelium of blood vessels. PAD4 was found in the lining and sublining layers of intact RA synovial tissue and diffusely in the necrotic tissue, but not in OA tissue. The diffuse PAD4 staining in the necrotic tissue could be due to release of PAD4 from disrupted neutrophils discussed in detail below, or alternatively unspecific binding of the antibody. Our results are in concordance with previous findings, where PAD2 and PAD4 were shown to be present in RA synovial tissue [3] and plastic adherent macrophage-like cells isolated from peripheral blood monocyte fraction [20]. PAD3 expression in the synovial tissue has not been shown previously but PAD3 mRNA was found recently in synovial tissue, not in cultured synovial cells [21]. In our specimens, PAD3 staining was strongest in the macrophage-like synoviocytes in the lining layer of OA and lining and sublining layers in RA synovial tissue (Figs. 6 and 7) co-localizing with macrophage marker KP1 (Fig. 11). This could explain the earlier PAD3 mRNA finding [21], since inflammatory macrophages are not long-lived in cell culture. Peripheral blood monocytes and macrophage-like adherent cells isolated from peripheral blood monocyte fraction did not seem to express PAD3 in an earlier study [20]. This could indicate that in addition to differentiation to macrophages also inflammatory activation is needed for PAD3 expression. In our study, some PAD3 staining was also detected in synovial stromal cells with more fibroblast-like morphology (Figs. 7d and 9d). The exact role of PAD3 in RA context remains to be clarified, but interestingly, PAD3 involvement in calcium-induced cell death pathway in neural stem cells was shown recently [4]. This earlier study suggested a role for PAD3 as a regulator of cell death or survival in neural stem cells. A similar role in regulation of synovial growth could be an interesting phenomenon from the perspective of RA disease progression also. To verify this hypothesis, the role and effect of PAD3 expression and activation in inflamed tissue needs further studies on functional level.

Citrulline- and homocitrulline-containing proteins localized to the necrotic areas of rheumatoid nodule and synovial tissue, as shown here by F95 staining and earlier by staining with anti-modified citrulline antibodies [22]. It is notable, that these antibodies, do not make a distinction between these two structurally homological amino acids [19] and thus some of the earlier findings concerning citrulline may involve homocitrulline as well when immunological detection methods have been used. Here, we used HPLC to distinguish the two amino acids in hydrolyzed tissue samples. Some necrotic areas could be found in seropositive RA knee synovial tissues. Fibrinoid necrosis was absent in the seronegative RA and OA knee synovial tissues, where only low levels of citrulline and homocitrulline was found. The highest content of both were found in the seropositive RA metatarsal synovial tissues that were characterized by fibrinoid necrosis (Figs. 1 and 2). In our data, the immunostaining results were consistent with the HPLC analysis showing staining by F95 antibody as well as PAD2, PAD3, PAD4 and myeloperoxidase (Figs. 8 and 9). The reliability of immunostaining of necrotic tissue is always a challenge and any conclusions should be considered critically. Therefore we prepared homogenates of necrotic tissue showing the binding of chemically modified citrulline- and homocitrulline-binding antibodies (KS350) to high molecular weight citrullinated or homocitrullinated proteins in this tissue. Digestion of DNA from the sample released some of the citrulline- or homocitrulline-containing proteins. Finding myeloperoxidase by Western blotting (Fig. 4), and in the histological sections of the necrotic area (Figs. 8 and 9), suggests that indeed neutrophils are involved in the seropositive RA chronic inflammation.

Myeloperoxidase is an enzyme found in granules of intact neutrophils. When an object is too big to be ingested, neutrophils use their chromatin and the enzymes from their granules as the last available defense mechanism of forming extracellular traps (NETs) [23] and these have been suggested as sources of citrullinated autoantigens [7]. Histone citrullination by PAD-enzyme releases the chromatin packing [24]. RA neutrophils are prone to form NETs. NET components have been found in RA blood [25] and synovial fluid and active PAD enzymes [26] in synovial fluid. Also increased levels of myeloperoxidase have been found in RA sera and synovial fluid [27, 28]. We suppose that in seropositive RA synovium neutrophils respond to the massive inflammatory reaction or presence of histiocytes in and around the necrotic area. Myeloperoxidase staining could be found in the middle of the necrotic area and also in the intact tissue adjacent to the necrotic area. In the necrotic tissue, these distinct defense mechanisms target and modify proteins at the site. Our present data suggests, that myeloperoxidase from neutrophil granules is present at active sites of inflammation in seropositive RA synovial tissues and it could be from extracellular traps or secreted. Detection of NETosis in tissue specimens is a challenge. However, these events can create a situation where citrulline and homocitrulline are present simultaneously in large amount in host proteins that are further presented to the inflammatory cells. This could be a part of the process for RA autoantibody induction, and at least partly explain the wide variety of antibody specificities that can be found in RA sera. Overall, our data supports the idea of repeated auto-vaccination as a propagator of RA [29].

In relation to RA, myeloperoxidase could also have an independent role in autoantibody development. The levels of thiocyanate are elevated in blood of smokers [30] and myeloperoxidase facilitates homocitrulline formation through a chemical reaction originating from thiocyanate [31]. Antibodies binding to carbamylated proteins have been found in RA sera and these were also suggested to be related to joint damage [9]. Here we found high levels of antibodies binding to citrulline- and homocitrulline-containing collagen telopeptides. As shown before [10], the presence of these antibodies was overlapping with ACPA positivity, but was not in clear relation with tissue citrulline and homocitrulline levels. Here it should be noted that not all of the sera analyzed were taken at the time of the operation and thus the antibody titers may not directly reflect the situation at the time of the operation. In general, the levels of these antibodies were highest in the seropositive RA knee and MTP subgroups that showed also the highest local tissue levels of citrulline and homocitrulline. Thus, the presence of high levels of citrulline- and homocitrulline-containing proteins in ACPA and carbamylated antibody-positive individuals could lead to persistence of inflammation leading to progression of tissue damage. The chemical protein modifications are still not enough for antibody induction as shown recently [32], but probably also genetic predisposition is needed.

In clinical perspective, our data suggests that there are distinct processes in RA and OA synovial tissue that have differences even between anatomical locations. In the necrotic tissue, the altered self-proteins are presented to the inflammatory cells, inducing antibody response that could cause secondary inflammation in other joints leading to chronic inflammation and tissue erosions. The removal of the necrotic tissue is the only way of extinguishing the local inflammatory process that the necrotic tissue upholds. In RA, the local and systemic control of inflammation is disturbed. These findings however raise new questions on pathophysiology behind the necrotizing events in RA synovium and the clinical possibilities on how to prevent these events. Moreover, since in RA the body is not able to remove all necrotic tissue that upholds the inflammation, surgery might indeed still be needed, until the mechanism behind the formation of necrotic lesions is fully understood and curable by other means. For surgeons, this means that synovial biopsies should be taken while performing synovectomy and if necrosis is seen this suggests that the patient could benefit from more extensive surgery. Removal of the necrotic mass and activated synovial tissue could be a key element in alleviating the local symptoms of the disease.

Some study limitation issues need to be taken into account while interpreting these results. The RA patient tissues were collected from patients with a long-lasting disease with severe tissue alterations in the joints and there is a selection bias, since all patients had to undergo surgery. The patients’ history of smoking and the medication in use are confounding factors and the tissue piece used for analyses only presents a small area of the synovial tissue. In addition, the method used here for citrulline and homocitrulline detection is semi-quantitative and based on a chemical modification reaction after hydrolysis of the tissue. Some citrulline or homocitrulline might be lost during hydrolysis and the chemical modification reaction is done individually to each sample, which may somewhat affect the result, so the absolute amounts of citrulline and homocitrulline could be somewhat greater than reported here, if a direct determination method was used. Despite these shortcomings, significant differences between groups could be detected and hence, the data is reliable albeit limited due to currently usable methodology.

## Conclusions

In conclusion, this study adds to the current knowledge by showing neutrophil enzyme myeloperoxidase in seropositive RA synovial tissue with homocitrulline. Disperse staining patterns of myeloperoxidase suggests NETosis or myeloperoxidase release from neutrophils. NETosis involves also peptidyl arginine deiminases and is a process creating citrullinated proteins released in the extracellular space. These findings can further explain the RA progression and disease mechanism.

## Key messages

- Citrullination and homocitrullination are present simultaneously in necrotic RA synovial tissue.
- Myeloperoxidase is found in seropositive RA synovial tissue and not in seronegative RA or OA.
- Myeloperoxidase and necrosis are more frequent in the metatarsal synovial tissues compared to knee synovial tissues.
- These results suggest neutrophil involvement in seropositive RA synovial inflammation.

## Abbreviations

ACPA: anti-citrullinated protein/peptide antibodies
CRP: C-reactive protein
DMARDs: disease-modifying antirheumatic drugs
HPLC: high-performance liquid chromatography
Ig: immunoglobulin
MTP: metatarsophalangeal
NET: neutrophil extracellular trap
OA: osteoarthritis
PAD: peptidylarginine deiminase
RA: rheumatoid arthritis
RF: rheumatoid factor
TNF-α: tumor necrosis factor alpha
